# Clinical Characteristics and Outcomes of Bloodstream Infections Caused by Metallo-β-Lactamase–Producing Enterobacterales in Argentina: A Subanalysis of the EMBARCAR Prospective Multicenter Cohort Study

**DOI:** 10.1093/ofid/ofag124

**Published:** 2026-04-08

**Authors:** Ezequiel Cordova, Juan Pablo Balbuena, Analia Mykietiuk, Javier Farina, Marcelo Gañete, Pablo Scapellato, María Inés Lespada, Esteban Nannini, Rosa Contreras, Eleonora Cunto, Laura Barcelona, Fernando Pasteran, Claudia Salgueira, Martín Hojman, Miriam Blanco, Horacio Lopez Alegre, William E Cox Fernandez, Cecilia Ezcurra, Darío Godoy, María A Biglia, María José Lopez Furst, Gustavo Mendez, María F Aguirre Rios, Javier Altclas, César Sanchez, Martin E Stryjewski

**Affiliations:** Infectious Diseases Unit (EC, MIL) and Department of Medicine (CS), Hospital Cosme Argerich, Buenos Aires, Argentina; Department of Research and Infectious Diseases Service, Instituto Médico Platense, La Plata, Argentina; Department of Research and Infectious Diseases Service, Instituto Médico Platense, La Plata, Argentina; Infectious Diseases Service, Hospital de Alta Complejidad Cuenca Alta Nestor Kirchner, Cañuelas, Argentina; Infectious Diseases Service, HIGA Evita Lanús, Lanús, Argentina; Infectious Diseases Service, Hospital Santojanni, Buenos Aires, Argentina; Infectious Diseases Unit (EC, MIL) and Department of Medicine (CS), Hospital Cosme Argerich, Buenos Aires, Argentina; Infectious Diseases Service, Sanatorio Británico, Rosario, Argentina; Infectious Diseases Service, Hospital Marcial Quiroga, San Juan, Argentina; Intensive Care Unit, Hospital F J Muñiz, Buenos Aires, Argentina; Infectious Diseases Service, Hospital Bernardo Houssay, Vicente López, Argentina; Antimicrobials Service, National Reference Laboratory for Antimicrobials, Department of Bacteriology, Instituto Nacional de Enfermedades Infecciosas, ANLIS “Carlos G. Malbrán”, Buenos Aires, Argentina; Infectious Diseases Service and Infection Control, Sanatorio Anchorena Recoleta, Buenos Aires, Argentina; Infectious Diseses Unit, Hospital Gral. De Agudos “Bernardino Rivadavia”, Buenos Aires, Argentina; Microbiology Laboratory, Hospital El Cruce, Florencio Varela, Argentina; Infectious Diseases Service, HIGA Presidente Perón, Avellaneda, Argentina; Infectious Diseases Service, Sanatorio Privado San José, Buenos Aires, Argentina; Infectious Diseases Service, Hospital Alemán, Buenos Aires, Argentina; Microbiology Laboratory, Hospital El Cruce, Florencio Varela, Argentina; Infectious Diseases Service, Hospital Quemados “Dr. Arturo Illia”, Buenos Aires, Argentina; Infectious Diseases Unit, Sanatorio Julio Méndez, Buenos Aires, Argentina; Department of Medicine, Division of Infectious Diseases, Hospital Escuela de Agudos Dr. Ramón Madariaga, Posadas, Argentina; Infectious Diseases Service, Hospital César Milstein, Buenos Aires, Argentina; Infectious Diseases Service and Infection Control, Sanatorio Trinidad Mitre, Buenos Aires, Argentina; Infectious Diseases Unit (EC, MIL) and Department of Medicine (CS), Hospital Cosme Argerich, Buenos Aires, Argentina; Department of Medicine, Centro de Educación Médica e Investigaciones Clínicas “Norberto Quirno” (CEMIC), Buenos Aires, Argentina

**Keywords:** aztreonam, bloodstream infection, ceftazidime-avibactam, enterobacterales, metallo-β-lactamase

## Abstract

**Background:**

Bloodstream infections (BSI) caused by metallo-beta-lactamase (MBL)-producing Enterobacterales (E) are associated with high mortality. However, in most Latin American countries there is a lack of clinical and microbiological data on BSI caused by MBL-E.

**Methods:**

This preplanned subgroup analysis of the multicenter, observational, prospective EMBARCAR study focused on adult patients with BSI caused by MBL-E in Argentina. Logistic regression adjusted by propensity scores (PS) for the treatment with ceftazidime-avibactam plus aztreonam (CAZ/AVI plus ATM) were used to identify variables associated with mortality.

**Results:**

A total of 140 patients with BSI caused by MBL-producing Enterobacterales were included. Median age was 59 years (IQR 46–70), and most patients were male (61%). Most frequent comorbidities were diabetes 23%, class III obesity 19%, and chronic renal failure 19%. Two thirds of patients were hospitalized in critical care units, 60% required mechanical ventilation and 38% presented with shock. Most frequent isolated microorganisms were *Klebsiella pneumoniae* (75%) and *Serratia marcescens* (12%). Most patients (64%) received combination therapy and 23% received CAZ/AVI plus ATM. Thirty-day mortality was 41%. In the multivariate PS adjusted analysis, INCREMENT-CPE score ≥8 was associated with increased risk of mortality (odds ratio [OR] 3.63; 95% CI: 1.13, 11.7), while CAZ/AVI plus ATM therapy with a lower risk of death (OR 0.18; 95% CI: .05, .58).

**Conclusions:**

In summary, BSI due to MBL-producing Enterobacterales resulted in a high mortality rate in our country. The use of CAZ/AVI plus ATM was associated with a decreased mortality. Better access to these antibiotics should be granted.

## Teaser

In this multicenter prospective study from Argentina, bloodstream infections caused by metallo-β-lactamase–producing Enterobacterales showed high mortality. The combination of ceftazidime-avibactam plus aztreonam was independently associated with lower mortality, supporting its potential as an effective therapeutic option in this setting.

Carbapenemase-producing Enterobacterales (CPE) have emerged as a major public health concern. Among them, metallo-β-lactamase–producing Enterobacterales (MBL) represent a particularly challenging group because of their broad-spectrum resistance profile and the scarcity of effective antimicrobial agents [1]. Bloodstream infections (BSI) due to MBL-producing Enterobacterales are associated with high morbidity and mortality [1, 2]. Importantly, regional differences have been reported in both the genetics of microorganisms and the clinical outcomes associated with these infections [3]. In addition, emerging real-life studies have suggested better outcomes with the use of new β-lactamase inhibitors (eg, ceftazidime-avibactam) associated with aztreonam to treat patients infected with MBL strains [2, 4]. In this scenario, generating high quality local data from prospective studies focused on the role of the new BLI-aztreonam combination in patients with BSI due to MBL-producing Enterobacterales is crucial.

The EMBARCAR study (Estudio Multicéntrico de Bacilos Resistentes a Carbapenemes en Argentina) was a prospective, multicenter cohort, conducted to describe the epidemiology and outcomes of BSIs due to carbapenem-resistant Gram-negative bacilli (CR-GNB) in Argentina. While the primary analysis provided insights into the overall burden of CR-GNB BSI and particularly in those patients with *Klebsiella pneumoniae*, a specific analysis on patients with BSI due to MBL-E was lacking [5]. This pre-planned subanalysis was conducted to describe the clinical characteristics, management, and outcomes of patients with BSI due to MBL-producing Enterobacterales in Argentina.

## METHODS

EMBARCAR was a multicenter, prospective, observational study conducted in Argentina between July 2020 and March 2022, which included patients with a positive blood culture for CR-GNB. Methods, including clinical variables, definitions, microbiological procedures, and ethical aspects, have been previously described in detail elsewhere [5]. Given the focus of the study on epidemiology and mortality, resolution of clinical parameters was not captured within the EMBARCAR study. For this subanalysis, we only included patients with a positive blood culture for MBL-producing Enterobacterales from 22 healthcare centers in Argentina. Patients were excluded if they had a previous blood culture with the same organism within 15 days, polymicrobial bacteremia, or if they did not receive any antibiotic treatment for the episode. Severity of illness at baseline was assessed using the INCREMENT-CPE score, with a score <8 considered mild-to-moderate and ≥8 as severe.

Identification of microorganisms and antimicrobial susceptibility testing (AST) were performed at each participating center according to CLSI recommendations [6] using the routine, validated methodologies available at each laboratory. Accordingly, AST results could be generated by disk diffusion, MIC determination using broth microdilution–based panels (eg, Sensititre), and/or automated AST platforms (eg, Vitek, Phoenix, MicroScan), with interpretation based on CLSI criteria or National Reference Laboratory for Antimicrobial Resistance (NRL, ANLIS/Malbrán, Argentina) recommendations [7]. In vitro synergy between aztreonam and avibactam was evaluated using the rapid agar prediffusion method endorsed by the NRL [8]. In addition, a subset of strains from the participating centers was sent to the NRL for confirmation of carbapenem resistance and for molecular detection of carbapenemase genes (bla_KPC, bla_NDM, bla_OXA-48-like, bla_IMP, bla_VIM) by multiplex PCR [9].

*In vitro* active therapy was defined as treatment with at least 1 in vitro active drug. Monotherapy and combination therapy were defined according to the number of in vitro active drugs administered; for the purposes of this subanalysis, CAZ/AVI plus ATM was considered monotherapy, despite the redundancy of ceftazidime. The primary outcomes of this subanalysis were to describe the epidemiology and clinical outcomes of patients with MBL-E BSI in Argentina; to identify risk factors associated with 30-day all-cause mortality; and to compare outcomes between patients treated with CAZ/AVI plus ATM and those treated with other active antibiotics.

Statistical analyses included descriptive statistics, Kaplan–Meier survival curves, and logistic regression to determine independent predictors of 30-day mortality. Given the nonrandomized administration of CAZ/AVI plus ATM, a propensity score (PS) was calculated using a multivariable logistic regression model with such treatment as the dependent variable. Covariates were clinically selected based on their known or suspected association with the outcome and included sex, age ≥60 years, Charlson score ≥3, and INCREMENT-CPE score ≥8. We used nearest-neighbor matching (1:1) without replacement to balance the treatment and control groups based on their propensity scores. The final multivariable model was adjusted by the PS. Kaplan–Meier survival curves were created according to antibiotic treatment with CAZ/AVI plus ATM before and after PS matching using a nonstratified log-rank test. Analyses were conducted in Stata version 12.0 (StataCorp, College Station, Texas). In order to address survivor bias a sensitivity analysis was conducted excluding patients who started CAZ/AVI plus ATM after day 3. Considering the possible different management across centers, a second sensitivity analysis using a fixed-effects Firth bias-reduced penalized-likelihood logistic regression model with hospital-specific intercepts was also performed. The study adhered to the principles of the Declaration of Helsinki and was approved by the research ethics committee of the Instituto Médico Platense (La Plata, Province of Buenos Aires), the Joint Commission for Health Research of the Province of Buenos Aires, and the ethics committees of all participating institutions. Based on the noninterventional nature of the study and the use of anonymized data, the requirement of informed consent was waived by the ethics committees. This study was completely designed, conducted, and funded by the Argentine Society of Infectious Diseases.

## RESULTS

Among 317 patients with BSI due to carbapenem-resistant Enterobacterales in the EMBARCAR study, 151 (48%) were infected with MBL producer strains. Among these patients, 11 were excluded due to the absence of antimicrobial treatment, resulting in a final cohort of 140 unique patients with BSI caused by MBL-producing Enterobacterales. The median age was 59 years (interquartile range [IQR], 46–70), and 61% of patients were male. The most frequent comorbidities were diabetes (23%), class III obesity (19%), and chronic renal failure (19%); almost half of the patients (48%) had a Charlson comorbidity index ≥3. COVID-19 at admission or during hospitalization was reported in 39%. The median time from admission to BSI episode was 23 days (IQR, 11–37). At the time of bacteremia, 66% of patients were in critical care units, 60% required mechanical ventilation, and 38% presented with shock. The median SOFA score was 5 (IQR, 2–8), and the median INCREMENT-CPE score was 8 (IQR, 6–12). Table 1 displays the demographics and clinical characteristics of patients according to 30-day vital status.

**Table 1.** Clinical Variables Among 140 Patients With Bloodstream Infection Due to Metallo-β-lactamase–Producing Enterobacterales According to 30-Day Vital Status

Variable	Global n = 140	Survivors n = 82	Nonsurvivors n = 58	*P*
Age, median (IQR)	59.0 (46.0–70.0)	58.0 (46.2–69.0)	62.0 (46.8–71.8)	.30
Male sex	86 (61.4%)	52 (63.4%)	34 (58.6%)	.60
Comorbidities				
Diabetes mellitus	32 (22.9%)	16 (19.5%)	16 (27.6%)	.31
Class III obesity	27 (19.3%)	13 (15.9%)	14 (24.1%)	.28
Chronic kidney disease	27 (19.3%)	16 (19.5%)	11 (19.0%)	>.99
Heart failure	13 (9.3%)	5 (6.1%)	8 (13.8%)	.15
Solid cancer	12 (8.6%)	6 (7.3%)	6 (10.3%)	.55
Hematologic Cancer	7 (5.0%)	1 (1.2%)	6 (10.3%)	.02
COPD	5 (3.6%)	3 (3.7%)	2 (3.4%)	>.99
HIV	6 (4.3%)	3 (3.7%)	3 (5.2%)	.69
Cirrhosis	7 (5.0%)	3 (3.7%)	4 (6.9%)	.45
Autoimmune disease	7 (5.0%)	1 (1.2%)	6 (10.3%)	.02
Solid organ transplant	10 (7.1%)	9 (11.0%)	1 (1.7%)	.046
Bone marrow transplant	2 (1.4%)	0 (0.0%)	2 (3.4%)	.17
Charlson comorbidity score, median (IQR)	2.0 (1.0–4.0)	2.0 (0.0–4.0)	3.0 (2.0–5.0)	.016
Previous hospitalization 90d	30 (21.4%)	18 (22.0%)	12 (20.7%)	>.99
Surgery in the last 30 d	28 (20.0%)	14 (17.1%)	14 (24.1%)	.39
Antibiotic therapy in the last 30 d	110 (78.6%)	66 (80.5%)	44 (75.9%)	.54
Carbapenem exposure (last 30 d)	58 (41.4%)	33 (40.2%)	25 (43.1%)	.86
Colonization by CR-GNB^a^	70 (50.0%)	45 (54.9%)	25 (43.1%)	.23
Time from admission to BSI episode (IQR), d	23.0 (11.0–37.0)	25.0 (13.2–41.0)	17.0 (10.0–34.8)	.14
Unknown source of infection	60 (42.9%)	41 (50.0%)	19 (32.8%)	.06
Documented source of infection	80 (57.1%)	41 (50.0%)	39 (67.2%)	.06
Central venous catheter	39 (27.9%)	20 (24.4%)	19 (32.8%)	.34
Pneumonia	11 (7.9%)	4 (4.9%)	7 (12.1%)	.20
Urinary infection	14 (10.0%)	12 (14.6%)	2 (3.4%)	.04
Surgical site infection	3 (2.1%)	2 (2.4%)	1 (1.7%)	>.99
Other documented source	73 (52.1%)	44 (53.7%)	29 (50.0%)	.73
Place of hospitalization^a^				
Emergency	3 (2.1%)	1 (1.2%)	2 (3.4%)	.57
General ward	44 (31.4%)	28 (34.1%)	16 (27.6%)	.46
Intensive care unit	93 (66.4%)	53 (64.6%)	40 (69.0%)	.72
Mechanical respiratory assistance^a^	84 (60.0%)	46 (56.1%)	38 (65.5%)	.30
Shock^b^	53 (37.9%)	22 (26.8%)	31 (53.4%)	.0025
INCREMENT-CPE mortality score, median (IQR)^b^	7.5 (6.0–12.0)	7.0 (6.0–10.0)	11.0 (6.2–14.2)	.0008
INCREMENT-CPE score ≥8^b^	70 (50.0%)	31 (37.8%)	39 (67.2%)	.001
SOFA Score, median (IQR)^b^	5.0 (2.0–8.0)	4.0 (2.0–7.0)	6.5 (4.0–10.0)	.001
COVID-19^c^	54 (38.6%)	34 (41.5%)	20 (34.5%)	.48
Laboratory^d^				
White blood cells (WBC) (mm^3^), median (IQR)	11.970 (8885–18 450)	11 675 (8900.0–15250.0)	14 600 (8555.5–22100.0)	.09
WBC ≥ 10 000/mm^3^	88 (62.9%)	50 (61.0%)	38 (65.5%)	.60
Platelets (mm^3^), median (IQR)	192 000 (122 750–298 000)	244 500 (147 750–334 000)	151 000 (92 750–230 750)	.0001
Creatinine (mg/dL), median (IQR)	1.1 (0.6–2.3)	0.9 (0.6–1.8)	1.3 (0.8–2.7)	.09
AST (U/L), median (IQR)	35.5 (25.0–57.8)	33.0 (24.0–56.0)	38.0 (25.0–62.0)	.29
ALT (U/L), median (IQR)	34.0 (15.0–63.0)	29.0 (14.2–55.5)	39.0 (18.0–72.0)	.22
Hematocrit (%), mean ± SD	28.2 ± 6.7	28.6 ± 6.7	27.5 ± 6.7	.40
Lactic Acid > 2 mmol/L	57 (40.7%)	30 (36.6%)	27 (46.6%)	.30
Corticosteroid therapy	53 (37.9%)	28 (34.1%)	25 (43.1%)	.29
Adequate empirical treatment	40 (28.6%)	23 (28.0%)	17 (29.3%)	>.99
Targeted treatment				
Includes CAZ/AVI plus ATM	32 (22.9%)	26 (31.7%)	6 (10.3%)	.0038
Other active antibiotics	108 (77.1%)	56 (68.3%)	52 (89.7%)	.0038
Combination therapy	89 (63.6%)	44 (53.7%)	45 (77.6%)	.0044
Monotherapy	51 (36.4%)	38 (46.3%)	13 (22.4%)	.0044
D until adequate treatment, median (IQR)	2.0 (0.0–3.0)	2.0 (1.0–3.0)	2.0 (0.0–3.0)	.46

^a^Documented CR-GNB colonization during the current admission or within the previous 12 m prior to the index blood culture.

^b^All clinical variables refer to the time of the index bloodstream infection.

^c^COVID-19 at admission or during the index hospitalization.

^d^Laboratory results at the time of the index BSI.

Abbreviations: BSI, bloodstream infection; COPD, chronic obstructive pulmonary disease; CR-GNB, carbapenem-resistant gram-negative bacilli; IQR, interquartile range.

The source of bacteremia was documented in 57% of patients, with central venous catheter (28%), urinary tract (10%), and respiratory tract (8%) as the most frequent sources. Compared with those who survived, patients who died had more often autoimmune diseases (1% vs 10%, p.02), hematologic cancer (1% vs 10%, p.02) and shock (27% vs 53%, p.0025). Patients who died also had higher median Charlson score (2 vs 3, p.016), SOFA (4 vs 6.5, p.001) and CPE score (7 vs 11, p.0008), respectively.

**Figure 1.**
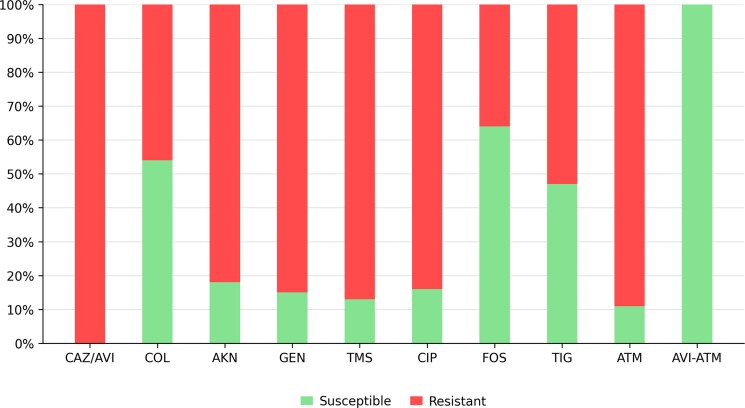
Antibiotic resistance profile of isolates from 140 patients with bloodstream infections caused by metallo-beta-lactamase–producing Enterobacterales. Abbreviations: CAZ/AVI, ceftazidime-avibactam; COL, colistin; AKN, amikacin; GEN, gentamicin; TMS, trimethoprim-sulfamethoxazole; CIP, ciprofloxacin; FOS, fosfomycin; TIG, tigecycline; ATM, aztreonam; AVI-ATM, in vitro synergy between avibactam and aztreonam.

The microorganisms isolated in this cohort were: *K. pneumoniae* 105/140 (75%), *Serratia marcescens* 17/140 (12%), *Providencia stuartii* 9/140 (6%), *Escherichia coli* 3/140 (2%), *Proteus mirabilis* 3/140 (2%), *Klebsiella aerogenes* 2/140 (1%), and *Enterobacter cloacae* 1/140 (1%). The antimicrobial susceptibility profile of MBL-producing Enterobacterales isolates is displayed in Figure 1. Overall, resistance to most antibiotics was high. Susceptibility was most commonly observed for fosfomycin (64%), colistin (54%), and tigecycline (47%). All isolates tested showed *in vitro* synergy to the combination of avibactam and aztreonam.

Resistance mechanisms were characterized as follows: only MBL in 125/140 (89%), MBL plus OXA-48–like in 9/140 (6%) and MBL plus KPC in 6/140 (4%). A phenotypic profile consistent with MBL plus extended spectrum β-lactamase (ESBL) was observed in 92/104 (88%) isolates. A total of 93/140 (66%) MBL-producing strains were further analyzed by the National Reference Laboratory. A total of 92/93 strains (99%) were New Delhi metallo-β-lactamase (NDM) and the remaining strain (1%) was an imipenemase.

Empirical antibiotic treatment was administered in 99% of patients, and in 29% of patients such treatment was deemed to be appropriate. The median time to *in vitro* active definitive treatment was 2 days (IQR, 0–3). Regarding definitive antimicrobial treatment, 23% of patients received a treatment including CAZ/AVI plus ATM, (97% as monotherapy), whereas 77% of patients were treated with other active antibacterials. These other antibacterials included colistin in 52%, tigecycline 29%, or fosfomycin in 25% of patients, respectively (Supplementary Table 1). Among patients who received antibacterials other than CAZ/AVI plus ATM, 81% received combination therapy. There was no significant difference in baseline severity between treatment groups: the median INCREMENT-CPE score was 9 (IQR, 6–12) in patients treated with CAZ/AVI plus ATM versus 7 (IQR, 6–11) in those receiving other antibiotics (*P* = .99). The median duration of *in vitro* active definitive therapy was 10 days (IQR, 7–14). Compared with patients who died, those who survived received more frequently CAZ/AVI plus ATM (32% vs 10%, *P*.0038) (Table 1).

**Figure 2.**
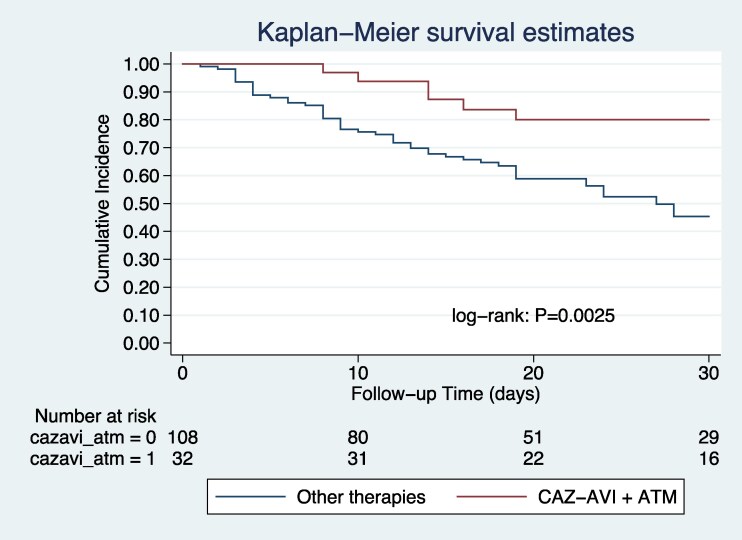
Survival curve among 140 patients with bacteremia due to metallo-beta-lactamase–producing Enterobacterales according to treatment with ceftazidime-avibactam plus aztreonam or other antimicrobials.

**Figure 3.**
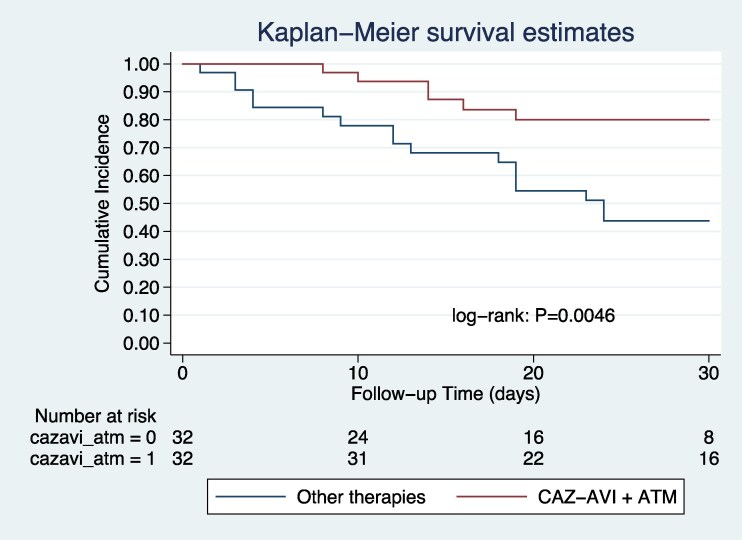
Survival curve among 64 propensity score-matched patients with bacteremia due to metallo-beta-lactamase–producing Enterobacterales according to treatment with ceftazidime-avibactam plus aztreonam or other antimicrobials.

In the multivariate analysis, an INCREMENT-CPE score ≥8 was independently associated with increased 30-day mortality (odds ratio [OR] 3.40; 95% CI, 1.64–7.05), while treatment with CAZ/AVI plus ATM was significantly associated with a reduction in mortality (OR 0.25; 95% CI, .09–.67). A propensity score-based matched analysis was carried out. A total of 32 pairs of patients treated with CAZ/AVI plus ATM and other active antibiotics were matched according to their propensity score. In the matched sample, both associations remained significant: INCREMENT-CPE score ≥8 (OR 3.63; 95% CI, 1.13–11.71) and CAZ/AVI plus ATM (OR 0.18; 95% CI, .05–.58). Table 2 displays the logistic regression before and after PS adjustment. Kaplan–Meier survival analysis also showed improved survival in patients treated with CAZ/AVI plus ATM, both in the unmatched (log-rank *P* = .0025; HR 0.30; 95% CI, .13–.69) (Figure 2) and matched cohorts (log-rank *P* = .0046; HR 0.29; 95%CI, .11–.73) (Figure 3). Both sensitivity analysis addressing survivor bias and Firth penalization confirm such findings. Details on the propensity score distribution, matching quality, covariate balance before and after matching, patient characteristics in the propensity score-matched cohort and sensitivity analyses are provided in the Supplementary material (Supplementary Table 2, Supplementary Figure 1 and Supplementary Figure 2, Supplementary Table 3, Supplementary Table 4 and Supplementary Table 5).

**Table 2.** Multivariate Analysis of 30-D Mortality in 140 Patients With Bacteremia Due to MBL-producing Enterobacterales

	Without PS Adjustment			With PS Adjustment		
	(n = 140)			(n = 64)		
Variable	OR	95% CI	*P* value	OR	95% CI	*P* value
INCREMENT-CPE score ≥ 8 points	3.40	1.64–7.05	.001	3.63	1.13–11.71	.031
Ceftazidime-avibactam + aztreonam	.25	.09–.67	.006	.18	.05–.58	.005

## DISCUSSION

This prospective, multicenter study in patients with BSIs caused by MBL-producing Enterobacterales provides several important findings.

The 30-day all-cause mortality rate in this cohort of patients with BSI due to MBL-producing Enterobacterales was high (41%). This result is consistent with previous studies from endemic regions, where death rates have ranged from 30% to 50% depending on illness severity, infection source, and adequacy of therapy [1, 2]. As an example, a recent multicenter trial emulation based on retrospective data in Argentina has recently reported an overall 44% mortality rate among 243 patients with BSI due to MBL-producing Enterobacterales [10]. Our prospective findings align with these reports, confirming that BSIs due to MBL-producing Enterobacterales carry a substantial mortality burden in our country. Importantly, in our study, severity of illness, as reflected by INCREMENT-CPE scores ≥8, was independently associated with increased mortality. This observation underscores the prognostic value of this score in our population of patients with BSI due to MBL-producing Enterobacterales [11].

Our cohort was predominantly composed of *K. pneumoniae* isolates (75%), with NDM being the most frequently detected MBL type (99% of characterized isolates). This epidemiological pattern reflects the global predominance of NDM-producing *K. pneumoniae*, particularly in endemic regions [12–14]. The resistance profiles revealed extensive drug resistance, with particularly high rates of resistance to aminoglycosides, quinolones, and trimethoprim-sulfamethoxazole. Remarkably, resistance to colistin was observed in almost half of the isolates (46%). While this resistance rate to colistin was similar to a recent report from Argentina [8], it was higher than resistance rates reported in other regions for MBL-producing Enterobacterales. These regional differences are most probably due to variation in resistance mechanisms including co-resistance patterns [15]. Importantly, nearly all isolates exhibited a phenotypic profile consistent with co-production of MBL and ESBL or KPC enzymes, which confers resistance to aztreonam. Nevertheless, in vitro synergy between CAZ/AVI plus ATM was observed in all tested isolates. This was in line with previous reports suggesting the combination of avibactam with aztreonam overcomes co-production of β-lactamases among MBL strains [13] [15, 16].

Another major finding of our study is the significant association between treatment with CAZ/AVI plus ATM and reduced 30-day mortality, even after adjustment for baseline severity and propensity score matching. The survival analysis using Kaplan–Meier curves further supported these observations, showing significantly improved 30-day survival in patients treated with CAZ/AVI plus ATM in both unmatched and matched cohorts. In addition, the sensitivity analyses addressing survivor bias and Firth penalization confirm our results. Importantly, these findings suggesting lower mortality in patients with MBL-producing Enterobacterales treated with aztreonam plus avibactam (with redundancy of ceftazidime) are consistent with observational studies and expert guidelines [3, 4, 17, 18]. Despite this potential benefit, access to CAZ/AVI plus ATM remains limited in many low- and middle-income countries, including many in Latin America [19]. Our study showed that only 23% of patients received CAZ/AVI plus ATM, largely reflecting barriers in access, particularly to CAZ/AVI. Ensuring availability of effective therapies is essential to improve outcomes in patients with multidrug-resistant Gram-negative infections [20, 21].

This study has several important limitations. First, it is an observational study and, although adjusted by propensity score and confirmed by sensitivity analyses, residual confounding cannot be excluded. However, given the scarcity of clinical trial data on infections caused by MBL-producing Enterobacterales, real-world evidence becomes particularly valuable in guiding clinical decision-making [22, 23]. Second, resistance testing was performed phenotypically and not confirmed by molecular methods in the reference laboratory in all cases, although this approach reflects real-world clinical practice. Most participating centers had access to rapid molecular or immunochromatographic assays to detect carbapenemase production directly from positive blood cultures (eg, multiplex PCR platforms or lateral-flow assays). However, because molecular microbiological workflows were not standardized across institutions, we did not systematically collect information on which rapid diagnostic tools were used at each site. For similar reasons, we were also unable to factor colonization into our models or determine its specific role in medical management. Third, the decision to administer CAZ/AVI plus ATM was not standardized and may have been influenced by clinician judgment, drug availability, and institutional protocols. Four, most of these patients had BSI due to *K. pneumoniae* and were included in our previous study [5]. Therefore, the current study should be interpreted with caution, basically as a subgroup analysis to confirm our previous findings in the group of patients with BSI due to MBL-producing Enterobacterales. Finally, as this study reflects a patient population from Argentina, the generalizability of its findings to other countries or regions should also be approached with caution.

In conclusion, BSI due to MBL-producing Enterobacterales are associated with high mortality in our country. The combination of CAZ/AVI plus ATM was independently associated with improved survival. Access to regimens containing aztreonam plus avibactam should be prioritized for patients with BSI due to MBL-producing pathogens. Improving such regional access to CAZ/AVI and aztreonam combinations could significantly impact the survival of these patients in Latin America.

## Supplementary Material

ofag124_Supplementary_Data

## Supplementary Data

Supplementary materials are available at *Open Forum Infectious Diseases* online. Consisting of data provided by the authors to benefit the reader, the posted materials are not copyedited and are the sole responsibility of the authors, so questions or comments should be addressed to the corresponding author.
