# Supplementary material for: Clinical Characteristics and Outcomes of Bloodstream Infections Caused by Metallo-β-Lactamase–Producing Enterobacterales in Argentina: A Subanalysis of the EMBARCAR Prospective Multicenter Cohort Study
Source: Open Forum Infect Dis. 2026 Apr 8;13(4):ofag124. doi: 10.1093/ofid/ofag124 (PMC13069563; doi:10.1093/ofid/ofag124)
Supplement: ofag124_Supplementary_Data [file ofag124_supplementary_data.pdf]

MBL-EMBARCAR SUB STUDY - SUPPLEMENTARY DATA

Table S1. Definitive treatment regimens used in bacteremia due to MBL-producing Enterobacterales and associated 30-day mortality

Treatment regimen	*Episodes*	*30-day mortality*	
	**N**	**N**	**%**
Ceftazidime/avibactam + aztreonam	31	6	19.4
Colistin	11	3	27.3
Ciprofloxacin + meropenem	10	3	30.0
Colistin + tigecycline	10	5	50.0
Colistin + meropenem	8	5	62.5
Colistin + fosfomycin	7	5	71.4
Colistin + fosfomycin + tigecycline	5	4	80
Other (<5 episode each)	58	27	46.6

Table S2. Evaluation of balance before and after matching (% bias)

Variable	Unmatched / Matched	Mean Treated	Mean Control	%bias	%reduct |bias|	t-test t	t-test p>|t|	V(T)/ V(C)
Sex	U	.6875	.59259	19.7		0.97	0.336	0.91
	M	.6875	.6875	0.0	100.0	0.00	1.000	1.00
Age ≥60 years	U	.25	.5463	−62.9		−3.02	0.003	0.77
	M	.25	.25	0.0	100.0	0.00	1.000	1.00
Charlson index ≥3 pts	U	.40625	.5	−18.7		−0.93	0.355	0.99
	M	.40625	.40625	0.0	100.0	0.00	1.000	1.00
Increment−CPE ≥8 pts	U	.4375	.51852	−16.1		−0.80	0.424	1.01
	M	.4375	.4375	0.0	100.0	0.00	1.000	1.00

* if variance ratio outside [0.49; 2.05] for U and [0.49; 2.05] for M

Sample	Ps R2	LR chi2	p>chi2	MeanBias	MedBias	B	R	%Var

```
-----------+----------------------------------------------------------------------
 Unmatched | 0.064      9.64     0.047      29.4       19.2       65.2*     0.73       0
 Matched   | 0.000      0.00     1.000       0.0        0.0        0.0      1.00       0
----------------------------------------------------------------------------------
* if B>25%, R outside [0.5; 2]
```

Figure S1. Visualization of distribution of propensity scores on treatment and control group

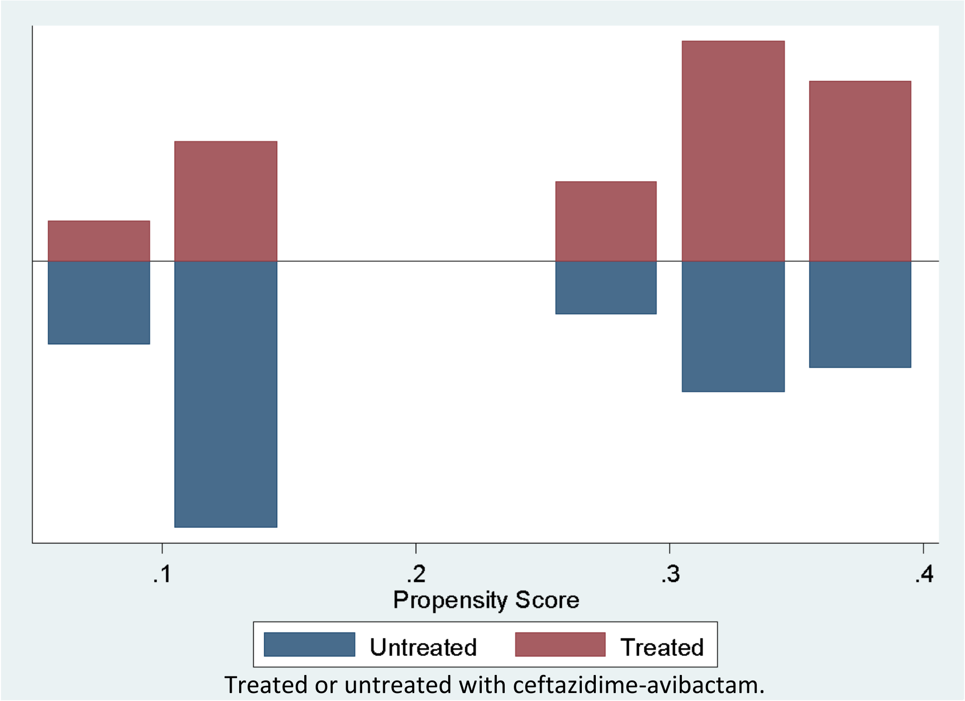

Figure S2. Visualization of matching quality assessment

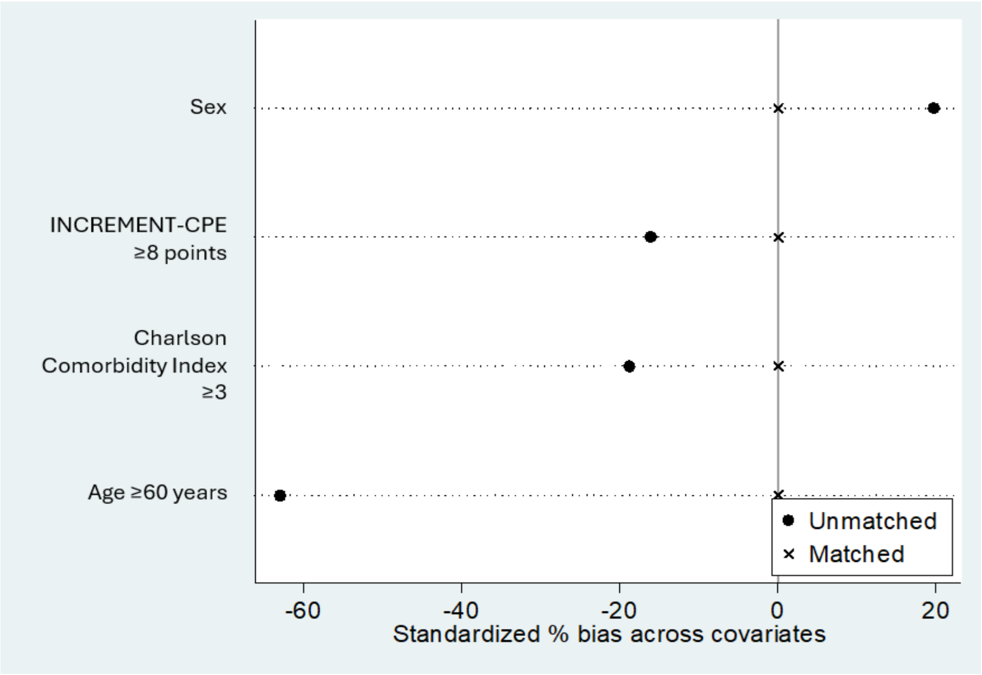

Table S3. Patient characteristics in the propensity score–matched cohort

Variable	Global<br>n=64	Other therapies<br>n=32	CAZ-AVI + ATM<br>n=32	P
Age, median (IQR)	57 (44-66)	58 (41-70)	56 (48-60)	.36
Male sex, n (%)	39 (60.9)	17 (53.1)	22 (68.8)	.2
Charlson score ≥3, n (%)	26 (40.6)	13 (40.6)	13(40.6)	1.0
Diabetes mellitus, n (%)	17 (26.6)	9 (28.1)	8 (25.0)	.78
Chronic kidney disease, n (%)	15 (23.4)	10 (31.3)	5 (15.6)	.14
Class III obesity, n (%)	14 (21.8)	13 (40.6)	1 (3.1)	< .001
Previous hospitalization 90 days, n (%)	13 (20.3)	6 (18.8)	7 (21.9)	.76
Antibiotic therapy last 30 days, n (%)	49 (76.6)	22 (68.8)	27 (84.4)	.14
Carbapenem exposure last 30 days, n (%)	20 (31.3)	10 (31.3)	10 (31.3)	1.0
Surgery in the last 30 days	12 (18.8)	2 (6.3)	10 (31.2)	.01
INCREMENT-CPE score ≥8, n (%)	28 (43.8)	14 (43.8)	14 (43.8)	1.0
Colonization by CR-GNB	32 (50.0)	11 (34.4)	21 (65.6)	.01
Days from admission to BSI episode, median (IQR)	22 (10-40)	21 (10-40)	23 (12-41)	.8102

Table S4. Sensitivity analyses addressing survivor bias: multivariable analysis of 30-day mortality in patients with bacteremia due to MBL-producing Enterobacterales.*

Variable	Without PS adjustment (n=136)			With PS adjustment (n=56)		
	OR	CI95%	P	OR	CI95%	P
INCREMENT CPE ≥8	3.35	1.61-6.96	.001	3.17	0.95-10.63	.062
CAZ/AVI + ATM	.29	.10-.78	.015	.21	.06-.72	.013

* Including only patients who started CAZ/AVI + ATM during the first 3 days from de BSI

Table S5. Multivariable analysis of 30-day mortality using Firth bias-reduced logistic regression with hospital-specific intercepts

30-day mortality	OR	SE	P	95% CI	
INCREMENT-CPE ≥8	4.41	2.05	.001	1.77	10.96
CAZ/AVI-ATM	.15	.14	.05	.02	1.00
